# A four-stage process for intervention description and guide development of a practice-based intervention: refining the Namaste Care intervention implementation specification for people with advanced dementia prior to a feasibility cluster randomised trial

**DOI:** 10.1186/s12877-019-1275-z

**Published:** 2019-10-21

**Authors:** Catherine Walshe, Julie Kinley, Shakil Patel, Claire Goodman, Frances Bunn, Jennifer Lynch, David Scott, Anne Davidson Lund, Min Stacpoole, Nancy Preston, Katherine Froggatt

**Affiliations:** 1 International Observatory on End of Life Care, Faculty of Health and Medicine, Lancaster University, Lancaster, LA1 4YG UK; 2 St Christopher’s Hospice, 51-59 Lawrie Park Road, Sydenham, London, SE26 6DZ UK; 3 Lancashire Clinical Trials Unit, University of Central Lancashire, Preston, Lancashire PR1 2HE UK; 4 Centre for Research in Public Health and Community Care, University of Hertfordshire, College Lane, Hatfield, Hertfordshire AL10 9AB UK; 5 Patient Representative c/o The Alzheimer’s Society, London, UK

**Keywords:** Implementation, Dementia, Palliative care, Intervention, Trial, Consensus methods, Nursing homes

## Abstract

**Background:**

Some interventions are developed from practice, and implemented before evidence of effect is determined, or the intervention is fully specified. An example is Namaste Care, a multi-component intervention for people with advanced dementia, delivered in care home, community, hospital and hospice settings. This paper describes the development of an intervention description, guide and training package to support implementation of Namaste Care within the context of a feasibility trial. This allows fidelity to be determined within the trial, and for intervention users to understand how similar their implementation is to that which was studied.

**Methods:**

A four-stage approach: a) Collating existing intervention materials and drawing from programme theory developed from a realist review to draft an intervention description. b) Exploring readability, comprehensibility and utility with staff who had not experienced Namaste Care. c) Using modified nominal group techniques with those with Namaste Care experience to refine and prioritise the intervention implementation materials. d) Final refinement with a patient and public involvement panel.

**Results:**

Eighteen nursing care home staff, one carer, one volunteer and five members of our public involvement panel were involved across the study steps. A 16-page A4 booklet was designed, with flow charts, graphics and colour coded information to ease navigation through the document. This was supplemented by infographics, and a training package. The guide describes the boundaries of the intervention and how to implement it, whilst retaining the flexible spirit of the Namaste Care intervention.

**Conclusions:**

There is little attention paid to how best to specify complex interventions that have already been organically implemented in practice. This four-stage process may have utility for context specific adaptation or description of existing, but untested, interventions. A robust, agreed, intervention and implementation description should enable a high-quality future trial. If an effect is determined, flexible practice implementation should be enabled through having a clear, evidence-based guide.

## Background

Palliative and end-of-life care interventions in care homes for people living with and dying from dementia will always be multi-faceted and context sensitive. This requires interventions to be carefully developed, tested and implemented [1–4]. However, experience shows that innovations can be recommended, adapted and implemented without this measured approach, with the flawed implementation of the Liverpool Care Pathway a cautionary tale for those working in palliative care and beyond [5]. An example of an innovative intervention that has had rapid uptake in care homes is Namaste Care, a multi-component approach to care for people with advanced dementia. Interventions in this field are important, as care for people with advanced dementia is usually provided in long term care settings, and these are likely to become the main place of death in the future [6]. Developed as a response to a lack of active care being offered to people with advanced dementia it has a philosophy based on person centred, holistic care [7, 8]. However, early findings on how and why it does (or does not) work are only just beginning to emerge [9].

Practitioner engagement and attitude and ‘fit’ of an intervention are known to have a major effect on adoption of innovation [10], and Namaste Care appears to have such an intuitive ‘fit’ with practitioners. Implementing evidence-based practice in nursing care homes is complex, with issues such as being on ‘common ground’, connecting with practice, and reconciling new practice with other priorities affecting change [4, 11]. Namaste Care resonates with practitioners because of its context sensitive, innovative, and effective approach to care for an overlooked resident group [12–14]. Evidence from small scale, qualitative or uncontrolled studies indicates an effect on symptoms such as agitation [15, 16] and behavioural symptoms [17]. Qualitative studies identify that staff recognise positive features of the intervention such as providing sanctuary, connections and community, calmness and vision [9, 18–20]. Problems implementing and sustaining the programme do, however, exist. Adjusting to the routines of Namaste Care can be difficult, and workforce turnover and management disruption endemic in long-term care can be barriers to both implementation and sustainability of the intervention [9, 21]. It is likely that the label ‘Namaste Care’ is applied to a wide variety of activity, and implemented in different ways [22]. The requirement for robust evaluation of effectiveness has been recognised, as there are no controlled, comparative trials of this intervention [9].

The challenge for any study of Namaste Care is that the intervention already exists in practice, albeit without sufficient evidence of effect. This is not a novel problem, health and social care practitioners are adept at identifying areas of care need and devising and implementing potential solutions that have little underpinning empirical evidence [23]. Healthcare practices, without evidence of effect, have been categorised in three ways: those that are known not to work, those where the evidence of effect is uncertain, and those in development or implemented without evidence [24]. Whilst the field of de-implementation is developing in order to assist the reduction or cessation of use of interventions known not to work, be unproven, or harmful [25], there is less attention paid to how best to test complex interventions that have already been organically implemented in some areas of practice, but where robust evidence is absent.

A particular challenge in a situation where a broadly defined intervention has already started to be implemented in practice is that of intervention description. A clearly specified intervention is required for a number of purposes including training, understanding fidelity, ascribing outcomes to the intervention, future replication, cost effective and appropriate implementation [26]. The Medical Research Council guidance on developing and testing a complex intervention focuses on intervention development (identifying the evidence base, identifying or developing theory, and modelling process and outcomes) and acknowledges that a common failing is inadequate description of the intervention [1]. The guidance requires a full description of the intervention, and an understanding of its components, so that it can be delivered during the evaluations, allowing for (and understanding) any flexibility and variation, and so that others can implement it outside the study. Understanding the components of an intervention is also important in understanding how the intervention works: what are the ‘active ingredients’ of an intervention and how do they exert their effect [27]?

Implementation scientists also focus on the importance of intervention description. It is recognised that an intervention can have interacting components: ‘core components’ (the essential and indispensable elements of the intervention) and an ‘adaptable periphery’ (adaptable elements, structures, and systems related to the intervention and organisation into which it is being implemented) [2, 28]. Intervention over specification should be avoided, to enable variation to fit different contexts, recognising the impossibility of describing every component of a complex intervention [29]. However, compared to knowledge on how to evaluate and implement interventions, there is relatively little guidance on how to develop and describe an intervention in a way that might maximise likely effectiveness [30, 31]. There is a gap in knowledge for those testing effectiveness of practitioner developed and implemented interventions. In these situations the intervention may have been differently understood, frequently adapted, and may differ from the original intent of those initiating the intervention [22]. Its theoretical underpinnings may be absent or not clearly articulated. It is unlikely that it has been carefully specified or adapted for a particular culture or context.

Potential ‘top down’ and ‘bottom up’ problems also exist. First, trial interventions can be challenging to incorporate in to day-to-day practice [32–34]. In the care home situation there are particular issues with conducting research including factors such as time constraints, staff turnover and low education levels [4, 35, 36]. In specifying this intervention for research purposes, it was important that it remained relevant to practice, and did not take on features known to affect implementation. Second, interventions developed from practice do not always reflect the intervention encountered in practice. For example, the aim of the Liverpool Care Pathway was to take excellent hospice care principles and embed them in acute hospital practice. However, the intervention as specified (the paperwork developed), did not reflect the knowledge, skills and attitudes required for its safe and appropriate use [37].

The aim of this paper is to present a four-stage model to refine an existing Namaste Care intervention and develop an intervention description, guide and training package to support a feasibility trial of the Namaste Care intervention. The four stages include collation of existing materials, exploring comprehensibility with staff who do not have experience of the intervention, using nominal group techniques to refine and prioritise the intervention and its format, and refining with our patient and public involvement panel.

## Methods

The overall aim of the study is to establish the feasibility of conducting a cluster randomised controlled trial of Namaste Care in a nursing care home context in the UK [38]. This is a phased research study involving the development of programme theories of how the Namaste Care intervention achieves particular outcomes and in which circumstances; developing an evidence-based Namaste Care intervention description and training package; and a feasibility cluster randomised controlled trial with embedded process and economic evaluations. Phase one (programme theory development) involved a realist review process [39]. This paper reports on phase two work as an exemplar of a method of developing and refining an intervention that has some existing practice presence, using SQUIRE 2.0 as the basis for reporting [40]. The research team included nurse academics, a research practitioner who had implemented Namaste Care, the trial manager, and patient and public involvement (PPI) representatives.

We planned four iterative stages to this phase of the study, with co-design of the intervention description with nursing care home staff and family carers central to the methods chosen (see Table 1).

**Table 1:** Stages in developing the intervention and implementation description, manual and training package

Developing and refining the intervention and implementation specification, manual and training package	
Stage one	Collecting and collating existing materials used to support the Namaste Care intervention. This incorporates using both best evidence on guideline development and results from the realist review to collate a draft intervention description.
Stage two	Exploring the readability, comprehensibility and utility of the emergent Namaste Care Trial Manual with nursing care home staff who did not have experience of Namaste Care.
Stage three	Using modified nominal group techniques with research team members, nursing care home staff and family carers who have experience of Namaste Care in practice. The aim was to present the findings of the realist review and factors that shape the intervention delivery; to refine and prioritise the implementation process for the delivery of the Namaste Care programme based on the realist review findings; and to inform the format of the Namaste Care programme and implementation and training resources.
Stage four	Presenting the programme guide, implementation resources and training package to the study patient and public involvement panel for final refinement prior to use in the feasibility trial.

Lancaster University Faculty of Health and Medicine Research Ethics Committee granted approval for this phase of the study (17 Nov 2016/FHMREC16028).

### Stage one methods: developing an initial draft intervention description and manual from existing Namaste Care materials

Existing materials used to support Namaste Care programmes in practice were requested and collated. Key contacts within the UK using or publishing about Namaste Care were approached, many identified by online searches of grey literature and/or their self-identification of use on publicly accessible websites, together with snowball methods to identify nursing care homes or other care institutions (e.g. hospices) known to be using or who have used Namaste Care in any form in the past. Written requests were sent to 69 identified organisations (2 UK NHS, 11 Hospice, 56 Nursing/Care Homes). The request asked if they would be happy to provide any written materials they have used to support the implementation of Namaste Care, with explicit information provided about the purpose of the request and study.

These materials were used to prepare a draft intervention and implementation description and manual. Emerging findings from our realist review [39] were used to prioritise components of the intervention, where the evidence for these components affecting people with advanced dementia were strongest.

The design of the draft manual version one was guided by current evidence on writing manuals and clinical guidelines [41–46]. This evidence was summarised as key principles used throughout the study to guide the presentation of materials about the Namaste Care intervention, that they be simple, consistent, organised, natural, clear and attractive. These are summarised in Table 2.

**Table 2:** Key design principles used to format the intervention specification manual

Clarity	
• Specific information about what to do, when and how.	
• Effective language including active verbs that specify a recommended action by whom, when, under what conditions, and with what level of obligation (must, should, may ….)	
• Avoid ambiguity when a term is vague or can be interpreted in more than one way (e.g. frequently, periodically)	
• Direct writing style and active voice	
• Proper punctuation with short sentences	
• Minimise abbreviations, hyphenations, jargon	
• Capture main idea with first few words so readers can skim text easily	
• Keep units of meaning together, using bulleted lists to deal with repetition or complex paragraph structures	
Persuasiveness	
• Crisp and persuasive messages.	
• Frame recommendations as ‘gain’ rather than ‘loss’	
• Focus on errors of omission (not doing the right thing) rather than commission (doing the wrong thing).	
Format – Multiple versions of documents	
• Multiple formats or alternate versions can influence accessibility and ease of use. Provide one page summaries.	
• Tailor guidelines to their intended end-users. Integrated into the way they do things.	
• Present them in ways that can be read and understood	
Format – Components	
• Key features that have most significance should be highlighted and differentiated from other recommendations	
• Use short summaries and algorithms. Flowcharts can describe stepwise recommendations for care, mimicking a real patient encounter.	
• Present most pertinent information concisely	
• Present information in an expected and logical order	
• Mimic familiar documents such as care plans or policy documents etc.	
• Don’t mix positive and negative instructions	
Format – Layout	
• Pictures on left and text on right	
• Use information visualisation through graphics and information display (e.g. tables, algorithms, pictures) and information context (framing, vividness, depth of field)	
• Left justification enables natural reading. Avoid italics or all upper-case text. 12 point font at least.	
• Bundling. Three bundles of three items easier to remember than nine items	
• Words used for procedural information and abstract concepts. Images used for special information, and detail. Tables can improve information clarity.	
• Colour – use primary colours	
• Strong contrast with background	
• Use distinctive visual characteristics for different elements	
• Purposeful use of highlighting, colour coding, boxes and bullets.	
• Colour code related graphics and text.	

Principles drawn from [41–47]

### Stage two methods. Exploring the readability, comprehensibility and utility of the emergent Namaste Care trial manual with nursing care home staff who do not have experience of providing Namaste Care

We invited nursing and support staff from two UK nursing care homes where Namaste Care had never been provided to participate in an informal two-hour workshop. These were a convenience sample of homes typical of those who provide care to those with advanced dementia. Potential participants received written information about the study prior to attendance, and written consent to participate was obtained before the workshop commenced. Materials were supplied to those unable to attend for any written feedback. The workshop was facilitated by two investigators (CW and KF) with an informal discussion on the overall format, style and content of the booklet, with written notes and agreements captured by the investigators. Participants were encouraged to write or draw on the materials which were retained for analysis. The analytic focus was on understandability and utility for those unfamiliar with the intervention.

### Stage three methods. Modified nominal group techniques with nursing care home staff and family carers who have experience of Namaste Care in practice

Two one-day consensus workshops took place, one in the north and the second in the south of England. The aim of the nominal group work was to present the findings of the realist review and factors that shape the intervention delivery; to refine and prioritise the implementation process for the delivery of the Namaste Care programme based on these findings; and, to inform the format of the Namaste Care programme and implementation resources.

#### Population

Nursing care home staff (includes managers, nurses, care assistants, activity coordinators or volunteers) from homes with experience in implementing Namaste Care. Family members/carers with experience of caring for people with advanced dementia who have experienced the Namaste Care programme.

*Inclusion Criteria:*

I. The nursing care home has current or previous experience of using Namaste Care in practice.
II. Managers, nurses, care assistants, activity coordinators or volunteers who have worked in a nursing care home setting for at least 6 months which currently uses or had used Namaste Care.
III. Family members of people with dementia: may be currently a family member for a person with dementia, or have held that role previously.
IV. Family members able to understand and communicate in English.

### Sampling and recruitment

#### Staff and volunteers

Nursing care homes from different provider types (private (corporate and owner managed) and not-for-profit) were sought through public knowledge (e.g. information on their websites) of those using Namaste Care, contacts with Namaste Care trainers, and advertising via our institutional websites and social media channels (e.g. anonymised twitter handles). A snowball approach was used so that those recruited were asked to identify other homes that may meet the inclusion criteria. An invitation letter was sent to care home managers who were asked to send a workshop invitation letter and participant information sheet to individual staff. Staff who indicated a willingness to participate were sent further details of the event. Out of pocket expenses to attend were reimbursed to all participants, and family members and volunteers reimbursed for their time. Letters of thanks were sent to nursing homes.

#### Family member recruitment

An invitation letter and participant information sheet was sent to all family carers identified by the care home manager as having had relatives who were receiving or had previously received the Namaste Care intervention in the nursing care home and met the inclusion criteria. Following receipt of a response slip, or having contacted the researcher, family members received details of the event.

### Modified nominal group methods

Modified nominal group methods included exposure to stimulus materials (written materials from step 2 sent 2 weeks ahead of the workshop and findings from realist review presented by CW via a 10 min power point presentation at the workshop), silent generation of ideas onto individual post-it notes, and sharing ideas as a round-robin and group discussion using and moving post it notes on large flip chart paper to clarify and rank elements of the intervention [50–53]. Participants were asked to consider the components of the intervention to support the delivery of Namaste Care into nursing care home practice; the relative importance of different elements; and adaptations required to the content of Namaste Care resources and implementation guidance in terms of language, style, appropriateness to the care context and presentation format.

#### Data collection and analysis

Comprised notes taken during the meeting and documents (e.g. silent generation of ideas on post-it notes and ordering and prioritisation on flip chart sheets) generated by participants in the meeting. These were summarised and circulated to participants by email for agreement on the decisions arising from the event. Analysis considered the frequency of ranking components of Namaste Care alongside a thematic analysis of reasoning for preferences.

### Stage four methods. Presenting the programme guide and implementation resources to the study patient and public involvement panel for final refinement prior to use in the feasibility trial

Finally, before the materials were used in the feasibility trial the study patient and public involvement panel (*n* = 5) discussed and commented on the materials, facilitated by NP. Written comments on the materials were supplied by participants.

## Results

### Stage one

Materials were supplied only by hospice organisations (*n* = 3). These materials included training materials for Namaste Care activities, monitoring forms for the Namaste Care sessions and outcome tools used to ascertain the impact of the Namaste Care on participating residents. The Namaste Care Programme Toolkit (76 pages) written incorporating learning from a prior Namaste study was also provided [9, 17, 48, 49]. In addition we drew from the 2nd Edition of the book about Namaste Care developed by the programme initiator [8]. There was good agreement on the timing, style and content of a Namaste Care session as these were essentially summaries or interpretations of the Namaste Care book.

At the end of this stage we had prepared a 21-page booklet, incorporating the use of infographics (using the free software Piktochart™) to present key areas of information. These were the materials presented in stage two.

### Stage two

The stage two workshop was held at one of the nursing care homes, but due to a combination of workload and staff sickness only three members attended (1 care home manager, 1 support worker, 1 activity coordinator). None had personal experience of Namaste Care in that home or elsewhere. Participants emphasised the utility of brief overview documentation, materials to enable family carers to understand the intervention, and the importance of graphical display to enhance orientation to the materials. They amended some wording to suit a UK nursing care home situation, important as the programme originated in the US. An example is the use of the wording ‘personal care’. In the nursing care home context this equates to intimate care for example, washing or being helped to the toilet. This differentiation between personal and personalised care was important because the delivery of personal care in public spaces is deemed inappropriate by the Care Quality Commission who regulate care provision in nursing care homes. Staff proposed the term ‘pampering’ to describe the Namaste Care related activity. Following the workshop, the written materials were further refined. This included adding more graphical elements to replace text, colour coding the sections of the manual to ease navigation, and tabulating areas of text to break them up.

### Stage three

Seventeen participants took part in 2 consensus workshops (*n* = 15 nursing care home staff, 1 family carer, 1 volunteer). One workshop was held in a North-West England Care Home facilitated by CW and SP (*n* = 3 participants from 1 nursing home 40 miles distant), the second in a London Hospice facilitated by CW, JK and SP (*n* = 12 participants, from three nursing home groups within a 40 mile radius). Key elements of Namaste Care had been presented in three sections: What is Namaste Care, Preparing the Namaste Care space and The Namaste Care Session. Following the first consensus workshop, an additional section was identified: Preparing people and organisations for Namaste Care. This was then fed back to, and ratified as important by, the attendees at the second workshop.

Elements presented as important in the silent generation of ideas and group discussion around what Namaste Care is emphasised the importance of person-centred care and making connections:

*‘Reaching the spirit within the person. The person may seem to have disappeared, but they ARE STILL THERE. NAMASTE finds them’. ‘Namaste care is the loving care for these people who are unable to participate with group activities’. ‘Dignified, loving, human to human* *connection* *. [emphases in originals] (Flip chart notes ‘What is Namaste’ sessions).*

The importance of preparing the home and space was considered in a number of different elements including training, record keeping, and assessment:

*‘Finding the right place and moment’. ‘Namaste should be in a* *peaceful* *environment’. ‘Not too much paperwork, simple’. ‘Include Namaste as part of induction training for new staff’. ‘To liaise with families and carry out* *individual risk assessments* *with each resident’. [emphases in originals] (Flip chart notes’ Getting your home ready for Namaste Care sessions)*

Participants discussed the flexibility of the Namaste Care sessions, reflecting on seasonal changes they had made (e.g. beach related activities in Summer), but identified what they felt to be core elements:

*‘Important to ask residents daily as each day is different’. ‘To greet residents to Namaste room and make sure they are comfortable enough’. ‘Serve fluids throughout the session to keep them hydrated’. ‘Gentle face wash, hairbrush with communication’.* *‘Feedback* *to family members’*. [emphases in originals]. Flip chart notes ‘The Namaste Care session’)

Other important changes included renaming the materials as a ‘guide’ rather than ‘manual’ to acknowledge the flexible, yet boundaried, nature of the intervention. The guide booklet was shortened, and materials made more succinct. Issues such as intervention timing, frequency, focus and staffing requirements were further specified. It was recognised that it was important to capture the relational and philosophical aspects of the intervention in the training and the intervention guide.

The intervention guide was used as the basis for training materials to support implementation in the care homes. Participants also helped identify potential adverse events that may be associated with the intervention.

### Stage four

**Fig. 1:**
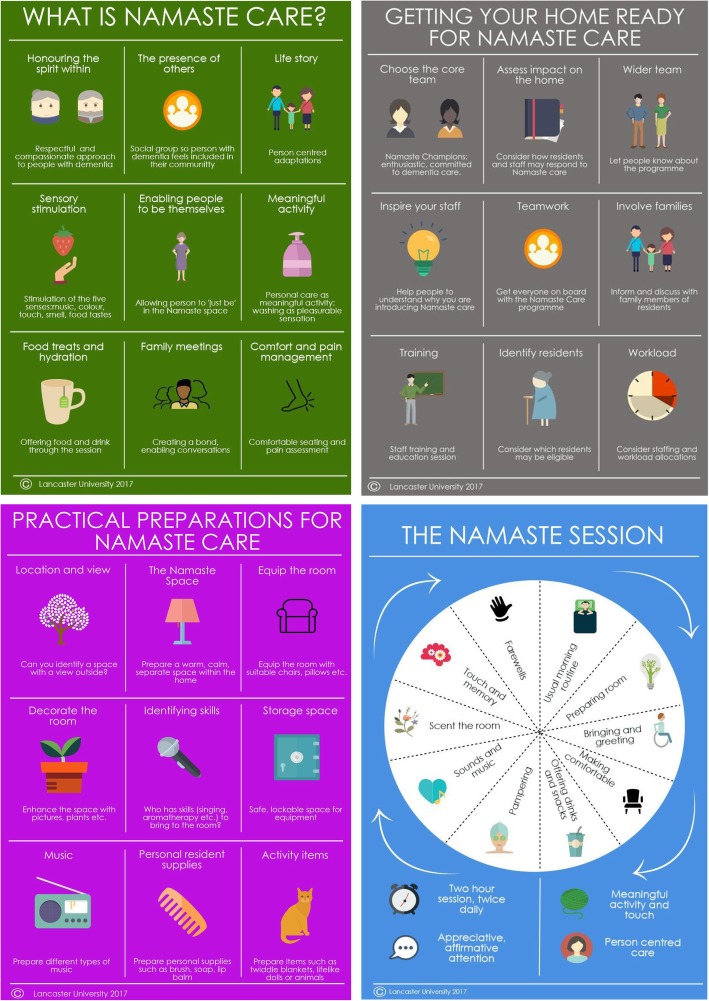
Infographics ‘What is Namaste Care’, ‘Getting your home ready for Namaste Care’, ‘Practical preparations for Namaste Care’, ‘The Namaste session’

The Patient and Public Involvement Group made suggestions on clarification of wording and recommended changes to the colours of the infographics to enhance readability. The final infographics used to support the study are displayed in Fig. 1.

## Discussion

The four-stage process for describing and developing an existing practice-based intervention prior to further testing and implementation appears to have utility. We were able to describe succinctly the Namaste Care intervention in a 16-page A4 booklet in a way acceptable to the nursing care home context. This was supplemented by four A4 infographics summarising the main elements of the intervention in an easy to read and user-friendly format. The guide is colour coded (to match the infographics) and uses flow charts and graphics to facilitate the reader’s understanding of and engagement with, the materials. Training materials follow the same style and format. The guide specifies the boundaries of the intervention, and guides implementation, whilst retaining the flexibility both inherent in Namaste Care, and required in a pragmatic feasibility trial.

**Fig. 2:**
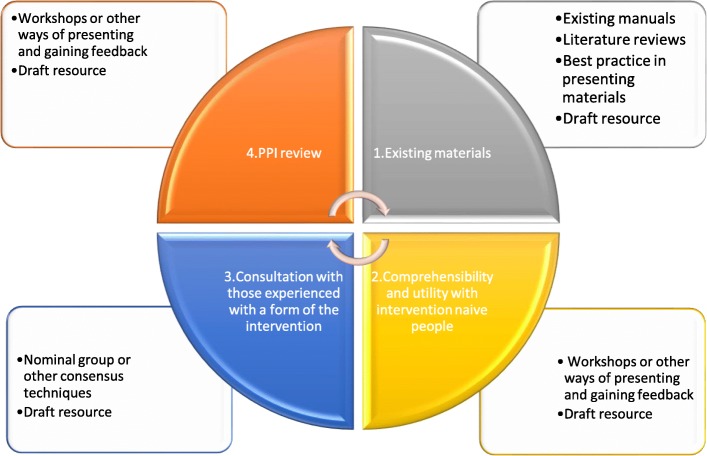
Four-stage process for describing and developing an existing practice based intervention

Intervention development is central to the Medical Research Council guidance on studying complex interventions—researchers are advised to consider whether they are clear about what they are trying to do, that the theoretical basis of the intervention has been used systematically to develop the intervention, and that it can be described fully [26, 54]. The Medical Research Council guidance is frequently used to optimise intervention development, but other frameworks such as intervention mapping, MOST (Multiphase Optimisation Strategy), the six steps in quality intervention development (6SQuID), and intervention modelling are also available [30, 55–58]. Although they use staged approaches which have similar features to the approach reported in our study (e.g. working with key stakeholders, involvement of patients and the public), these typically still are only used in novel intervention development [59]. The four-stage process used in this study to describe the intervention for research use may have utility for other researchers faced with similar challenges. These four stages are conceptually congruent with many frameworks for intervention development or implementation. For example, the Knowledge to Action Framework emphasises that resources should be produced in a collaborative fashion with end users and other interested parties [60], and this involvement was a key feature of the four step process described here. We propose that this four-stage process could be integrated as an additional component to existing frameworks for intervention development or implementation where there is a requirement for an existing intervention to be described, developed or refined. This generic process is presented in Fig. 2.

This four-stage process could, for example, be implemented in the development element of the Medical Research Council guidance for complex interventions [1], or the optimisation stage of MOST [57].

### Strengths and limitations of the study

The strengths of the study lie in the structured, inclusive and open approach to intervention refinement; opening the black box where many studies fail to describe fully either their intervention or its development. There was a clear relationship between the findings of the realist review [39] and the perceptions of those experienced in Namaste Care.

There were, however, challenges and potential biases that must be acknowledged. There were difficulties in engaging people throughout the process. Only hospice organisations provided information to stage 1, and it may be that the way they use or describe Namaste Care differs to nursing care homes. Few people took part in stage 2, although those who did were very engaged in the process and represented the key staff (nurses, activity coordinators and care support workers) expected to deliver such an intervention. Whilst we anticipated a larger attendance, pressures of day-to-day work in the context of staff sickness had to take priority. This is a reality of much engagement and consultation work with nursing care homes, especially where funds to replace staff were not available. We would recommend that those using this process in the future cost such funding into their processes.

As there is no known sampling frame of those using this intervention, recruitment of people into stage 3 had, by necessity, to involve informal procedures such as social media and word of mouth. This may introduce bias. In this instance a number of attendees had previously been involved in a similar training programme, which may have affected their responses in unknown ways. Few family carers or volunteers participated, although they were acknowledged as potentially important in intervention delivery, and their voices were captured in our PPI group in stage four. It may be that individual interviews at a place close to, or at, home could facilitate their involvement. Whilst we worked hard to ensure geographical diversity, many participants worked in or around London, and again this may introduce unknown biases due to particular difficulties of staffing nursing care homes in city areas where there is large turnover of staff and many may not have English as a first language. Consensus work may be challenging for some, privileging those who feel able to speak in such settings, or who have lower literacy levels. These issues were minimised through offering a variety of processes including silent, written, generation of ideas as well as small supportive table-based discussions that should enable all to have some form of participation.

### Recommendations for future use of this four-stage process

This process is likely to have utility across a number of studies, and we recommend its use in practice. However consideration should be given to a number of different aspects of the model that would benefit from critical adoption and enabling adaption of the process in the future.

- This process needs to be appropriately costed in to future research, including staff replacement costs and funding for a greater number of more local consensus meetings.
- Consideration should be given to how to further facilitate the involvement of lay people or family carers.
- Time needs to be allowed for this process, which took approximately 8 months due to the time taken to receive and process materials, and run three different forms of consultation and consensus work, alongside a comprehensive literature review process.
- Adaptation may be needed where it is anticipated that there are few written materials to support an existing intervention, and how the initial stimulus material could be generated.

## Conclusions

The four-stage process described here may have utility for researchers testing the effect of existing interventions, or where they need to adapt an existing intervention in a culturally or context specific way. Careful development and specification of an intuitively helpful intervention both enables an understanding of fidelity within the subsequent trial, but also facilitates future implementation, or indeed de-implementation. Future research could test these steps with other interventions, and report on its utility and development both in process evaluations of trials, in implementation studies, and in conjunction with other frameworks.

## Data Availability

The datasets used and/or analysed during the current study are available from the corresponding author on reasonable request.

## Abbreviations

6SQuID: Six steps in quality intervention development
MOST: Multiphase Optimisation Strategy
PPI: Patient and Public Involvement
